# Malformation artério-veineuse utérine: à propos de deux cas au Centre Hospitalier Universitaire de la Guadeloupe

**DOI:** 10.11604/pamj.2021.38.307.24924

**Published:** 2021-03-24

**Authors:** Mègnissè Sèna Lokossou, Gelly Akouala, Allyriane Aganahi, Mahoublo Vodouhe, Andrea Larissa Lokossou, Ambre Tramier, Gülen Ayhan, Eustache Janky

**Affiliations:** 1 Département de Gynécologie et d’Obstétrique, Centre Hospitalier Universitaire de Pointe à Pitre, Pointe à Pitre, Guadeloupe,; 2 Centre Hospitalier Universitaire Borgou-Alibori, Parakou, Bénin,; 3 Service de Radiologie, Centre Hospitalier de Blois, Mail Pierre Charlot, 41000 Blois, France

**Keywords:** Malformations artério-veineuses, diagnostic, traitement, rapport de cas, Arteriovenous malformations, diagnosis, treatment, case report

## Abstract

Les malformations artério-veineuses utérines sont une étiologie possible de métrorragies persistantes notamment en cas d´antécédent de fausses couches, de maladies trophoblastiques. Les auteurs rapportent les aspects diagnostiques et thérapeutiques de deux observations de malformations artério-veineuses utérines compliquant les suites du post-abortum à la maternité du Centre Hospitalier Universitaire de Pointe à Pitre en Guadeloupe. Les patientes ont présenté des métrorragies dans les suites d´un avortement traité par curetage. L´échographie couplée au Doppler a suspecté une malformation artério-veineuse. L´artériographie confirme le diagnostic et permet dans le même temps la réalisation d´un traitement conservateur par embolisation artérielle. Aucune complication n´est enregistrée. La méconnaissance des malformations artério-veineuse utérine peut avoir des conséquences délétères allant d´une hystérectomie d´hémostase par hémorragie cataclysmique au décès.

## Abstract (en)

Uterine arteriovenous malformations are possible causes of persistent metrorrhagia, in particular in patients with a history of abortion and trophoblast diseases. We here report the diagnostic and therapeutic features of two patients with uterine arteriovenous malformations complicating post-abortion in the Maternity Ward of the University Hospital of Pointe-a-Pitre in Guadeloupe. Patients had metrorrhagias after abortion with curettage. Arteriovenous malformation was suspected based on ultrasound combined with Doppler. Arteriography confirmed the diagnosis and allowed, in the same time, for conservative treatment by arterial embolization. No complication was reported. The lack of knowledge about uterine arteriovenous malformations can lead to deleterious consequences ranging from haemostatic hysterectomy due to cataclysmic hemorrhage to death.

## Introduction

Une malformation artério-veineuse (MAV) correspond à une communication anormale directe entre un réseau artériel et un réseau veineux, sans l´intervention d´un réseau capillaire [1]. Les malformations artério-veineuses utérines acquises (MAVU) sont peu évoquées dans le cadre de l´exploration d´une métrorragie persistante après une grossesse avec une issue défavorable (fausse couche, maladies trophoblastiques). Leur incidence réelle est mal connue, mais il s´agit a priori d´une situation très rare [1, 2]. Il faut y penser afin d´éviter la réalisation d´un curetage hémostatique qui serait inutile voire dangereux pour la patiente. Nous rapportons deux patientes présentant une malformation artério-veineuse intra-utérine dans le post-abortum dans le service de gynécologie-obstétrique du Centre Hospitalier Universitaire de la Guadeloupe. Les métrorragies étaient le signe fonctionnel constant; le diagnostic était suspecté à l´échographie Doppler confirmé par un scanner. Le traitement a reposé sur l´embolisation avec une évolution satisfaisante.

## Patient et observation

**Figure 1:**
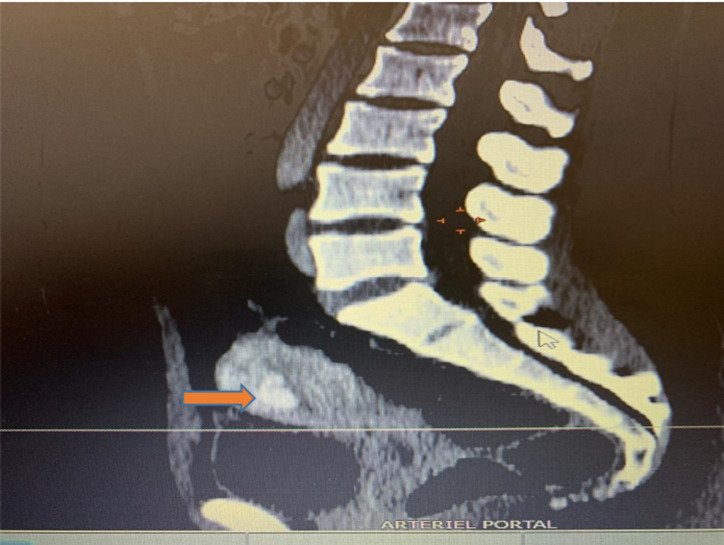
reconstruction sagittale médiane après injection de contraste (formation rehaussée tubulée utérine corporéale antérieure)

**Figure 2:**
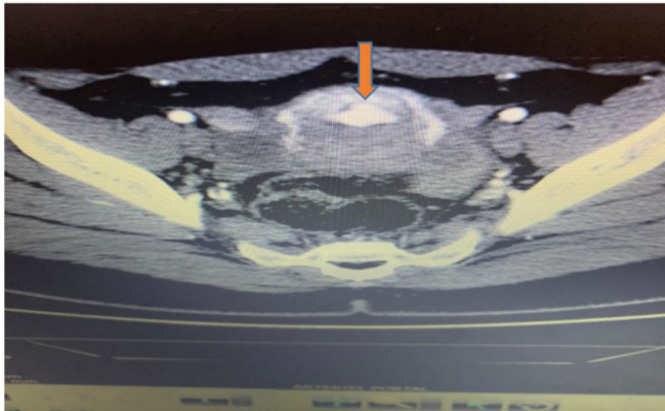
coupe axiale après injection de contraste (formation nodulaire rehaussée au temps artériel de l'injection faisant suspecter une malformation artério-veineuse)

Patiente N°1 âgée de 21 ans sans antécédents particuliers; gestité deux; parité un; ayant bénéficié d´une interruption volontaire de grossesse médicamenteuse par Misoprostol. Elle est admise à l´hôpital à un mois du post-abortum pour des métrorragies de grande abondance avec une hémodynamique stable. L´examen obstétrical note une origine endo-utérine du saignement avec un utérus de taille normal et mou au toucher vaginal. La gonadotrophine chorionique humaine bêta (BHCG) plasmatique était négative. A l´échographie Doppler, on retrouve quelques débris intra-utérins avec des images hypo-échogènes prenant le Doppler au niveau du fond vaginal. Le scanner pelvien conclut à une malformation intra-utérine avec un résidu anévrismal sous-endométrial associées à des varices pelviennes bilatérales (Figure 1, Figure 2). Une angiographie suivit d´une embolisation artério-veineuse est réalisée. Les suites étaient simples avec un arrêt des saignements.

**Figure 3:**
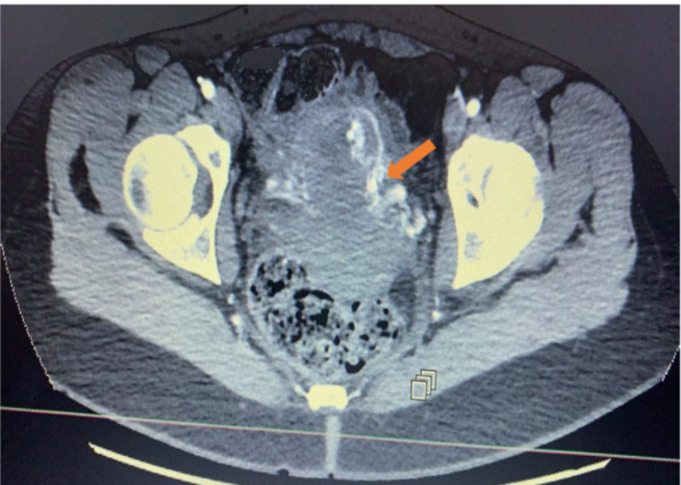
coupe axiale après injection de contraste (formations serpigineuses veineuses fortement rehaussées au temps artériel faisant évoquer une malformation artério-veineuse)

Patiente N°2 âgée de 35 ans présente dans ses antécédents une infertilité primaire avec des lésions d´endométriose; deux kystectomies per-cœlioscopique. Elle a bénéficié de deux tentatives de fécondation in vitro (FIV) dont la première a abouti à un échec et la seconde marquée par une cinétique anormale des BHCG et à l´échographie la présence, de deux sacs gestationnels sans vésicules vitellines sont visibles. Une interruption médicamenteuse de la grossesse a été réalisée avec un contrôle satisfaisant de la vacuité utérine au quinzième jour. L´évolution est marquée deux mois après par un tableau de méno-métrorragie. L´examen retrouve un saignement endo-utérin de grande abondance avec une anémie à 8,9 g/dl. Le BHCG plasmatique était négatif. A l´échographie Doppler, on retrouve une image anéchogène avec un flux au Doppler. Le scanner pelvien retrouve une malformation artério-veineuse du versant gauche du corps utérin avec extension endo-utérine et une probable extravasation du produit de contraste (Figure 3). L´artériographie hypogastrique sélective retrouve une malformation artério-veineuse latéralisée à gauche avec un drainage veineux vers la veine gonadique droite. Il n´y a pas d´anomalie de la vascularisation utérine droite. La patiente a bénéficié d´une embolisation à l´onyx de la malformation utérine gauche par voie radiale. Les suites étaient simples. Un contrôle par hystéroscopie est réalisé à distance et retrouve une cavité utérine normale.

## Discussion

Le terme de malformation artério-veineuse est souvent utilisé par excès dans les anomalies artério-veineuse. Leur incidence est mal connue. Il s´agit à priori d´une situation très rare mais potentiellement mortelle [1, 3]. Ces anomalies vasculaires utérines peuvent être responsables de méno-métrorragies chroniques mais aussi parfois de saignements cataclysmiques d´où l´intérêt de les diagnostiquer avant tout geste thérapeutique. Elles peuvent être congénitales ou plus fréquemment acquises. Les formes congénitales sont en rapport avec un défaut de développement vasculaire au cours de la vie embryonnaire [4]. Les formes acquises se rencontrent en général après un traumatisme utérin: curetage, révision utérine ou césarienne. Elles peuvent être également secondaires à une rétention de tissu trophoblastique, notamment suite à des grossesses môlaires [2]. Dans nos deux cas, la MAV est survenue au décours d´une interruption médicamenteuse de la grossesse. Le développement de la MAV s´explique comme étant consécutive à une rétention de cellules trophoblastique avec un défaut de régression de la vascularisation péri-trophoblastique [1].

Le diagnostic de MAV est évoqué devant des méno-métrorragies récidivantes, chez la femme jeune, mais parfois les MAVU peuvent se révéler par des douleurs pelviennes, des dyspareunies et/ou une anémie [1, 2]. Dans le cas de MAVU acquises, les ménorragies surviennent le plus souvent pendant les premières menstruations qui suivent le geste traumatique [5].

Trois examens paracliniques sont utiles au diagnostic des MAVU: l´échographie pelvienne couplée au Doppler couleur, l´imagerie par résonnance magnétique (IRM) et l´artériographie [2]. L´échographie Doppler montre des îlots anéchogènes, confluents, intra-myométraux, hypervascularisés, avec des vitesses artérielles systoliques très élevées [1, 6].

Dans notre étude, l´échographie Doppler suspectait la lésion, le scanner pelvien l´a confirmé et l´artériographie l´a visualisé. L´utilisation de l´hystéroscopie a été rapportée mais s´avère de réalisation difficile et peu contributive dans un contexte de saignement massif. Il a été décrit la visualisation de la MAVU sous forme d´une structure vasculaire bosselée pulsatile en surface de la cavité utérine. L´IRM est déterminante pour le diagnostic des MAVU. Son intérêt est double; d´une part, elle permet d´éliminer d´autres diagnostics tels que les lésions inflammatoires et néoplasiques utérines [2], d´autre part, elle permet une localisation géographique précise de la malformation en visualisant les vaisseaux nourriciers. L´angio-IRM en séquences rapides pondérées T1 est un examen de choix car il permet de réaliser une étude hémodynamique de la prise de contraste de la malformation [7]. Les difficultés d´obtention d´une IRM en urgence dans notre contexte expliquent sa non réalisation.

Les options de traitement dépendent de la pathologie sous-jacente et vont de l´abstention thérapeutique, à l´hystéroscopie opératoire, l´embolisation, la ligature des artères utérines voire l´hystérectomie. L´embolisation est pratiquée en première intention chez la plupart des patientes symptomatiques. Elle présente un taux de réussite élevé avec un taux de complications faible, comme le montre l´étude de Ghai *et al*. [8]. En effet sur 15 patientes, il obtient un taux de succès de 93% avec un taux de complications de 0,4%. Selon l´analyse de la littérature de Sanguin S *et al*. [2], les taux de succès à la 1^e^ tentative sont de 57%. L´embolisation peut être répétée en raison de l´échec à la première tentative. La résection hystéroscopique peut être une alternative conservatrice à l´hystérectomie chez les patientes désireuses de conserver leur fertilité [9]. L´embolisation est un examen invasif nécessitant des radiologues expérimentés et un plateau technique adapté. Il existe quelques complications peu fréquentes et non spécifiques aux MAVU. Il s´agit le plus souvent de complications mineures telles que des douleurs pelviennes temporaires [10]. Cependant, des complications graves bien qu´extrêmement rares ont été rapportées, et sont liées le plus souvent à une embolisation de l´artère iliaque interne créant des nécroses cutanées, des déficits neurologiques et des fistules recto-vésico-vaginales. Dans notre étude, aucune complication n´a été retrouvée.

## Conclusion

Les malformations artério-veineuses acquises doivent être évoquées devant l´apparition de métrorragies persistance dans le post-partum. Le diagnostic précoce par l´échographie pelvienne couplée au Doppler a permis un traitement conservateur par embolisation chez nos deux patientes.
